# Emerging Issues in Occupational Disease: Mental Health in the Aging Working Population and Cognitive Impairment—A Narrative Review

**DOI:** 10.1155/2020/1742123

**Published:** 2020-01-30

**Authors:** Gabriele Giorgi, Luigi I. Lecca, Jose M. Leon-Perez, Silvia Pignata, Gabriela Topa, Nicola Mucci

**Affiliations:** ^1^Department of Human Sciences, European University of Rome, Via Degli Aldobrandeschi, 190, 00163 Rome, Italy; ^2^Department of Experimental and Clinical Medicine, University of Florence, Largo Piero Palagi 1, 50139 Florence, Italy; ^3^Department of Social Psychology, University of Seville, Camilo Jose Cela s/n, 41018 Sevilla, Spain; ^4^Aviation Faculty School of Engineering, University of South Australia, Adelaide, SA, Australia; ^5^Department of Social and Organizational Psychology, National Distance Education University (UNED), Madrid 28040, Spain

## Abstract

Cognitive impairment has often been reported in scientific literature as a concern derived from chronic exposure to work-related stress. Organizational factors can contribute to the onset of this concern especially in a susceptible population such as elderly workers. The aim of our study was to review the last five years of scientific literature, focusing on experimental and epidemiological studies, possible mechanisms implicated in the onset of cognitive decline due to work-related stress, and the recent organizational strategies to prevent detrimental effects of stress on cognitive processes. A literature search was performed in scientific platforms Medline and Web of Science, by means of specific string search terms, restricting the search to the years of publication 2014–2019. Thirty-three articles were identified and qualitatively evaluated, reporting narratively the main point of interest. At this stage, six articles were excluded because they did not meet the inclusion criteria. Only a few articles considered the population of the elderly workers, often with a short follow-up period. Strategies to manage stress with organizational procedures are scarce. Mechanisms implicated in the development of cognitive impairment due to stress are not fully explained and seem to include a chronical decrease in the inhibitory process of neurological pathways. Further research that focused on strategies to manage stress in elderly workers, with the aim of preventing cognitive impairment processes, is warranted.

## 1. Introduction

Mental health in occupational settings is a major topic for occupational medicine, psychology, and social science [1, 2], with important implications for general health, psychological well-being, and company productivity [3, 4]. Since the decrease of classic occupational disease, due to the general improvement of working conditions, more attention is being paid to the mental well-being of employees. In the last five years, a consistent number of publications have been produced on this topic [5–8]. Modern industry 4.0 is changing the way of working, requiring high mental performance along with low physical effort. In fact, industry 4.0 represents the global trend towards automation and data exchange in manufacturing technologies. The term industry 4.0, often intended as a synonym of the fourth industrial revolution, include innovative processes such as the Internet of things (IoT), cyberphysical systems, and cognitive computing and artificial intelligence. Modern 4.0 factories are characterized by high connectivity between production lines and operators' control through a system that can visualise the entire production line and make decisions on its own.

This high technological scenario needs extensive adaptability of the workforce, and some population groups, such as elderly workers, could be more vulnerable to develop stress due to being less predisposed to change [9, 10].

The global aging of the working population could contribute to the problem that needs policy strategies to support and promote an active aging of the elderly population [11], considering also the occupational stress contribution to poor psychological and physical health, in terms of work-related stress, work-family conflict, shift work, and bullying behaviors [12, 13]. Since elderly people are per se more prone to develop a cognitive impairment due to their age advancing [14], the other risk factors that can accelerate this process may be prevented [15, 16]. A relationship between work-related stress and cognitive impairment has been recently reported in several scientific reports and literature reviews [17, 18]. Thus, there is sufficient evidence to consider work-related stress as a factor that is able to contribute to cognitive impairment. Since work-related stress could lead to cognitive impairment, its impact on a more susceptible population, such as elderly people, could be more pronounced. The role of occupational stress in the development of cognitive impairment and its impact on the elderly workforce have not been fully investigated, and the mechanisms implicated and organizational strategies to prevent the onset of stress and its consequences are not fully understood. The aim of this study was to investigate, through a narrative literature review restricted to the last five years, the relationship of work-related stress on cognitive impairment in the elderly working population, along with organizational preventive strategies and the pathophysiological causes of the mental decline attributable to high psychologically demanding work environments.

## 2. Materials and Methods

A literature review was performed in Medline and Web of Science databases. We considered articles published in the last five years, from 1 January 2014 to 31 March 2019. We selected articles published in the English language, including only studies performed with human samples. Following the PICO strategy for scientific research (population, intervention, comparison, and outcomes) [19], a specific string of search was used, including the most common search term for each PICO topic: Population, Intervention, Comparison, and Outcome.

Inclusion criteria were as follows:

- Study population: working population aged over 18 years
- Study design: cross-sectional studies, longitudinal studies with a follow-up case, case-control studies, and randomized clinical trials
- Exposure: work-related stress, occupational stress, job strain, job control, job support, and effort-reward imbalance
- Outcome: cognitive abilities measured in terms of errors, injuries, processing speed, alertness, distraction, memory, and testing of intellectual skills (e.g., intelligence)
- Publication type: articles in scientific journals

Exclusion criteria were as follows:

- Study population: working population less than 18 years old and animal samples
- Study design: reviews, meta-analyses, and case report studies
- Exposure: psychological stress related to caregiver activity, physical stress, chemical exposure, biological risk factors exposure, heat stress, and oxidative stress
- Outcome: short-term cognitive impairment and structural changes of the brain
- Publication type: letters to the editor, comments, abstracts, and book chapters

The following search terms were combined in several search term strings:

Population: workers, occupational group, working population, work, job, and job task. Exposure: stress, work-related stress, job strain, strain, job demand, job control, job support, effort, reward, effort-reward imbalance, organizational factors, and work organization. Outcome: cognitive impairment, cognitive effects, cognitive behavior, memory, and cognitive tasks.

A total of 214 articles were identified and listed to evaluate if they met the inclusion criteria. By means of a title-abstract screening, articles were defined as eligible for inclusion and then integrally read. Only articles that met the inclusion criteria were then included after a complete reading of the full text.

## 3. Results and Discussion

**Figure 1:**
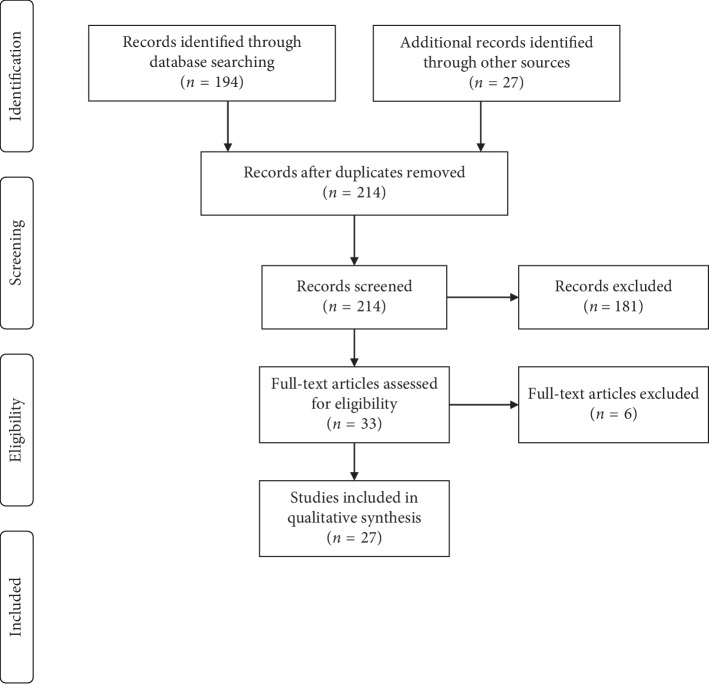
Flow diagram of the literature search strategy and review process, following PRISMA 2009 flow diagram rules.

Figure 1 shows a flow diagram of the literature search strategy and the review process following PRISMA 2009 flow diagram rules [20]. Twenty-seven articles that met inclusion criteria at the title-abstract reading stage were identified and evaluated.

A consistent number of studies about the relationship between occupational stress and cognitive impairment in the aging population were published. Recent research focused not only on the onset of dementia or Alzheimer's disease [21] but also on those subclinical changes in mental performance that can conduce a more important cognitive decline in elderly people.

A clinical case-control study performed by Eskildsen et al. [22] considered the relationship between work-related stress and the impairment in neuropsychological test performance, finding that self-reported stress patients had the worst performance in prospective memory, speed, and complex working memory, compared with controls without work-related stress. Despite this, the cross-sectional study design does not allow the inference about the causality of occupational stress affecting neuropsychological performances. Moreover, chronic cognitive impairment cannot be evaluated with a cross-sectional study design. The same authors repeated the assessment of neuropsychological performance in the same group of patients and controls after one year [23] and found that the former patients with prolonged work-related stress continued to perform worse than controls, with a significant impairment also in memory function. Despite the short follow-up of only one year, the study demonstrated that prolonged work-related stress could have a detrimental prolonged effect on cognitive function. Another study by Eskildsen and colleagues in 2017 investigated a change in cognitive impairments in midlife patients (mean age: 44 years) with work-related stress and found a partial association with a change in perceived stress and sleep disturbances over time [24].

Focusing on elderly people, two studies conducted by Sindi et al. in 2017 [21, 25] strengthen the hypothesis of an influence of work-related stress on cognitive performances. They found a significant association between higher levels of midlife work-related stress (mean age at the baseline of 50 years) and worse performance on global cognition and processing speed and a higher risk of mild cognitive impairment, dementia, and Alzheimer's disease in later life (mean ages at the reexamination phases of 71 and 78 years, respectively). The association was not seen after the extended follow-up possibly reflecting a critical time window for the effects of midlife stress. The long mean follow-up period (25 years) and the adequate sample size (2000 subjects at the initial phase) make the study a high-quality investigation. The importance of considering the subclinical effects of cognitive impairment due to work-related stress has to be considered in light of how this can impact the global population level, resulting in worse cognitive performance.

Similar evidence was reported by Agbenyikey et al. [26], who found that high job strain and low job control, at midlife, in a sample of 1429 Caucasian residents (median age at the baseline of 46 years) are associated with decline in verbal learning and memory, with a more evident association for the younger subgroup of participants (<65 years). Considering that, in the European Union (EU) Member States, the most general retirement age is 65 years [27], a more pronounced association exactly in this age class underlines the importance of this evidence in a population that is still working. Consistent with Sindi et al.'s study, the onset of cognitive impairment seems to have a window period in the youngest old instead of the oldest old.

On the other hand, a longitudinal study performed by Andel et al. [28] found that less job control and greater job strain were not significantly associated with change in episodic memory in the period leading up to retirement but were associated with significantly poorer episodic memory at retirement and an accelerated rate of decline in episodic memory following retirement. A longitudinal study performed by Sabbath and colleagues, published in 2016, deals with the problem of cognitive impairment in older age, by investigating which psychosocial work characteristics are able to predict changes in a cognitive function [29] of a sample aged more than 55 years. They argue, in an inconclusive manner, that low-control jobs during working life may be associated with impairments in cognitive function in early old age. These findings are in line with the aforementioned studies, adding new insight about how kinds of psychological work characteristics are more prone to promote a cognitive impairment, such as types of work characterized by a low decision latitude. Improving job control could be a valid strategy to slow down cognitive decline due to the combined effect of age and work stress.

Another longitudinal study performed by Rijs and colleagues in a population of employees aged 55–64, considering nonemployees as the control group, found that middle-aged workers are equally likely to experience memory complaints as nonworking age-peers, but on the other hand, among workers, those with cognitively demanding work were more likely to have memory complaints [30]. Jonsdottir and colleagues presented a case-control follow-up, testing the differences in the cognitive assessment of patients with stress-related exhaustion several years after they initially sought medical care [31]. They found that patients (mean age of 46 years at the baseline) still performed significantly poorer than controls (mean age of 50 years at the baseline) with regard to cognitive functions, suggesting a prolonged effect of work-related stress exhaustion that has to be taken into consideration at the moment of their return to work. In fact, a cognitive impairment could still be present at the resumption of the work activity, contributing to worse job performance. However, the small sample size limited the interpretation of results. Focusing on the postmenopausal women, in a study conducted in women aged between 45 and 66 years, negative correlations were observed between the majority of cognitive functions and the intensity of stress at work and the majority of factors that caused this stress [32].

Possible mechanisms implicated in cognitive impairment due to work-related stress exposure have been investigated by Landolt and colleagues [33]. Although the study was conducted in a small population of young workers between 16 and 24 years, the results suggest that a reduction in vagal activity seems to have a detrimental effect on reducing decision making and reaction time in subjects exposed to prolonged work-related stress. A study by Marshall and colleagues in a sample with a mean age of 68 years [34] suggested a role of stress on affecting elderly participants' inhibitory control in attentional and sensorimotor domains. Results of this study are in the same direction of research performed by Landolt and colleagues, supposing a reduction in vagal activity and inhibitory control as a mechanism able to affect cognitive performance, although the first was performed in a young population (mean age: 18 years), and the second in an elderly population (mean age: 68 years), considering as a control group a sample of young subjects (mean age: 21 years).

Sokka and colleagues conducted an experimental case-control study in 2016 [35] in a sample of 30 participants affected by burnout (mean age of 47 years) and observed a decrease in working-memory related P3b responses over the posterior scalp and an increase over frontal areas, with respect to the control group. These results suggest that burnout is associated with deficits in the cognitive control needed to monitor and update information in working memory. Boschi and colleagues found that a relationship between high cognitive disorganisation and high cardiopulmonary and anger scores as well as low perceived self-efficacy was associated with high cognitive disorganisation, suggesting a putative role of thinking patterns and state of mind on visceral factors. In particular, perception of what is stressful may lead to the body's stress activation pathways, which in turn may cause cognitive impairment [36]. Interactions between social and genetic risk factors have been investigated by Hasselgren who found a significant effect of work control on dementia risk only for men, along with a moderation effect of the major genetic risk factor for Alzheimer's disease, that is, apolipoprotein E allele [37]. These findings highlight the importance of such interactions between social and genetic risk factors to better understand multifactorial diseases such as dementia.

Some studies deal with the efficacy evaluation of a stress-management intervention, such as an aerobic training program or an individual cognitive behavior therapy, on improving cognitive performance in workers exposed to occupational stress, by means of a controlled randomized clinical trial. While aerobic training at a moderate-vigorous intensity within a multimodal rehabilitation program seems to improve episodic memory in patients (mean age of 42 years) with work-related exhaustion disorder [38], the combination of an individual cognitive behavioral therapy (CBT) with a brief workplace intervention fails to demonstrate a reduction in cognitive difficulties at any time point during a 10-month follow-up [39]. A possible contribution of a better work organization for the prevention of a cognitive decline in aging workforces was suggested by a longitudinal study performed by Riedel and colleagues [40]. They reported a positive association of balanced exchange between high effort and high reward at work with improved cognitive function over 6 years among a group of the middle-aged population (mean age of 44 years at the baseline), where the cognitive function was assessed by perceptual speed and verbal fluency. Further research aimed at clarifying the best strategies to improve cognitive performance impairment due to work-related stress is warranted.

In a cross-sectional study performed by Allan and colleagues [41], high occupational stress was related to more frequent failures of attention, memory, and concentration in telephone nurses (mean age of 41 years), with relevant consequences on the decision-making process. Due to the cross-sectional study design, it is not possible to make inferences about the direction of causality in the reported associations. Nevertheless, it is important to underline the possible impact of cognitive failure in job tasks with high responsibility, such as health care workers. These results suggest that burnout is associated with deficits in the cognitive control needed to monitor and update information in working memory. Similarly, Barbe et al. found a linkage between perceived stress, subjective concerns about cognitive function, and impairment of work function [42]. On the contrary, Lees and Lal did not find any association between stress and cognitive performance in a sample of middle-aged nurses with a mean age of 37 years [43].

A case-control study conducted by Golonka and colleagues deepened the psychobiological nature of burnout and its effect on cognitive impairment by means of an EEG monitoring during a specific error monitoring trial. They found emerging cognitive problems in the nonclinical burnout group with respect to the control group [44]. Once again, the mean age of participants was 36 years old. That middle age class does not allow these findings to be extended to older workers.

## 4. Conclusions

Our narrative review provides an update of the emerging problem of the decline of cognitive performance in elderly workers exposed to work-related stress. Despite the relevant number of studies published on the topic of cognitive impairment due to work-related stress, few studies were conducted in an elderly population of workers that is per se more prone to develop cognitive impairment, due to the multifactor causality of this problem. In conclusion, the present narrative review highlighted the importance of taking into consideration the subclinical detrimental effect of stress on the mental performance of workers exposed to prolonged work-related stress, especially in those jobs that require a high level of concentration and decision making resources. Moreover, some gender and genetic factors seem to play a relevant role in the complex linkage between work-related stress and cognitive impairment, underlying a multifactor genesis of cognitive outcomes including organizational, genetic, and behavioral factors. These findings can be useful to target improvement strategies aimed at enhancing the active aging of the population of the elderly workers. Further research is warranted to enhance our understanding of how a better organizational environment can improve the psychological and mental health of elderly workers.
